# Photobiomodulation and Coenzyme Q_10_ Treatments Attenuate Cognitive Impairment Associated With Model of Transient Global Brain Ischemia in Artificially Aged Mice

**DOI:** 10.3389/fncel.2019.00074

**Published:** 2019-03-19

**Authors:** Farzad Salehpour, Fereshteh Farajdokht, Javad Mahmoudi, Marjan Erfani, Mehdi Farhoudi, Pouran Karimi, Seyed Hossein Rasta, Saeed Sadigh-Eteghad, Michael R. Hamblin, Albert Gjedde

**Affiliations:** ^1^Neurosciences Research Center, Tabriz University of Medical Sciences, Tabriz, Iran; ^2^Department of Medical Physics, Tabriz University of Medical Sciences, Tabriz, Iran; ^3^ProNeuroLIGHT LLC, Phoenix, AZ, United States; ^4^Higher Educational Institute of Rab-Rashid, Tabriz, Iran; ^5^Department of Medical Bioengineering, Tabriz University of Medical Sciences, Tabriz, Iran; ^6^School of Medical Sciences, University of Aberdeen, Aberdeen, United Kingdom; ^7^Wellman Center for Photomedicine, Massachusetts General Hospital, Boston, MA, United States; ^8^Department of Dermatology, Harvard Medical School, Boston, MA, United States; ^9^Harvard-MIT Health Sciences and Technology, Cambridge, MA, United States; ^10^Departments of Clinical Research and Nuclear Medicine, Odense University Hospital, University of Southern Denmark, Odense, Denmark; ^11^Department of Neuroscience, University of Copenhagen, Copenhagen, Denmark; ^12^Department of Neurology & Neurosurgery, McGill University, Montreal, QC, Canada; ^13^Department of Radiology and Radiological Science, Johns Hopkins University, Baltimore, MD, United States

**Keywords:** transcranial photobiomodulation, Coenzyme Q_10_, aging, global ischemia, mitochondrial biogenesis, neuroinflammation, learning and memory

## Abstract

Disturbances in mitochondrial biogenesis and bioenergetics, combined with neuroinflammation, play cardinal roles in the cognitive impairment during aging that is further exacerbated by transient cerebral ischemia. Both near-infrared (NIR) photobiomodulation (PBM) and Coenzyme Q_10_ (CoQ_10_) administration are known to stimulate mitochondrial electron transport that potentially may reverse the effects of cerebral ischemia in aged animals. We tested the hypothesis that the effects of PBM and CoQ_10_, separately or in combination, improve cognition in a mouse model of transient cerebral ischemia superimposed on a model of aging. We modeled aging by 6-week administration of D-galactose (500 mg/kg subcutaneous) to mice. We subsequently induced transient cerebral ischemia by bilateral occlusion of the common carotid artery (BCCAO). We treated the mice with PBM (810 nm transcranial laser) or CoQ_10_ (500 mg/kg by gavage), or both, for 2 weeks after surgery. We assessed cognitive function by the Barnes and Lashley III mazes and the What-Where-Which (WWWhich) task. PBM or CoQ_10_, and both, improved spatial and episodic memory in the mice. Separately and together, the treatments lowered reactive oxygen species and raised ATP and general mitochondrial activity as well as biomarkers of mitochondrial biogenesis, including SIRT1, PGC-1α, NRF1, and TFAM. Neuroinflammatory responsiveness declined, as indicated by decreased iNOS, TNF-α, and IL-1β levels with the PBM and CoQ_10_ treatments. Collectively, the findings of this preclinical study imply that the procognitive effects of NIR PBM and CoQ_10_ treatments, separately or in combination, are beneficial in a model of transient global brain ischemia superimposed on a model of aging in mice.

## Introduction

Aging is a multifactorial process characterized by progressive decline of physiological functions and potential development of a wide array of diseases (López-Otín et al., 2013). Cognitive impairment is one of the most important aspects of aging brains (Harada et al., 2013). Mitochondrial dysfunction negatively affects neuronal metabolism by means of oxidative stress. Neuroinflammation is active in normally aging brains and is implicated in subsequent cognitive decline (Bishop et al., 2010; Khorrami et al., 2014). As a consequence of the limited availability of aged animals, and the costs of obtaining and housing an adequate number of such animals for experimental studies, natural aging models can be prohibitive. As sufficient natural aging is lengthy, rapid access to aged animals is a limiting factor of pre-clinical research. As an alternative approach, we modeled natural aging by chronic administration of D-galactose to rodents. D-galactose is a reducing hexose sugar that enters the brain and reacts with many cellular components at high concentration, producing oxidative damage to lipids and DNA (Ho et al., 2003; Sadigh-Eteghad et al., 2017). The chronic D-galactose treatment induces changes that resemble natural aging and mimics several characteristics of brain aging (Wei et al., 2005; Sadigh-Eteghad et al., 2017). The administration to rodents has been shown to cause neurocognitive impairments, including memory deficits, increased inflammatory responses and mitochondrial dysfunction (Shan et al., 2009; Sadigh-Eteghad et al., 2017; Salehpour et al., 2017).

Cerebral ischemia is a major risk factor during aging with consequences for the occurrence and severity of cognitive impairment (Chen et al., 2010). Cardiovascular events such as heart attacks and strokes are more common in older individuals, and the likelihood and severity of brain damage are correspondingly higher due to mitochondrial dysfunction. Therefore, age-related structural and functional changes potentially increase the vulnerability of the brain to ischemic insults (Ueno et al., 2002). During cerebral ischemia, the reduced blood flow and the subsequent lack of oxygen, glucose, and other nutrients disturb cellular homeostasis (Chen et al., 2011). Excessive production of reactive oxygen species (ROS) in mitochondria activates signaling pathways that ultimately result in further mitochondrial dysfunction and abnormal bioenergetics (Onyango et al., 2010; Olmez and Ozyurt, 2012). Transient bilateral common carotid artery occlusion (BCCAO) is a well-established experimental approach in rodents that exploits the adverse influence of acute cerebral hypoperfusion on cognitive mechanisms (Schiavon et al., 2014).

Due to the inverse relationship between the capacity for endogenous mitochondrial biogenesis and chronological aging, the ability of the brain to recover from an ischemic insult is much reduced in older individuals (López-Lluch et al., 2008). Upstream regulators of mitochondrial biogenesis, e.g., transcription factors such as SIRT1 (silent mating-type information regulation 2 homolog 1) and PGC1-α (peroxisome proliferator-activated receptor gamma coactivator-1 alpha) play key roles in the pathophysiology of brain ischemia (Chen et al., 2011). Down-regulation of markers of mitochondrial biogenesis such as NRF1 (nuclear respiratory factor 1) and TFAM (mitochondrial transcription factor A), as well as lower numbers of mitochondria, are observed in reperfusion injury after ischemia (Liu et al., 2014). In addition, decreased cerebral blood flow (CBF) yields pro-inflammatory responses such as production of tumor necrosis factor-α (TNF-α) and interleukin (IL) IL-1β from the ischemic endothelium which leads to larger infarction volumes and exacerbates brain injury (Anrather and Iadecola, 2016). In addition, NO produced by inducible NO synthase (iNOS) in activated microglia and damaged cerebrovascular endothelium is involved in the detrimental inflammatory response to an ischemic insult (Zoppo et al., 2000). The evidence suggests that mitochondria are the key targets both of ischemic injury and brain aging (Gjedde, 2005; Reddy, 2008). Here, we exploit the knowledge that the mitochondria are targets both of aging and of ischemic insults to the brain, with a potential for development of novel therapeutic approaches based on stimulation of mitochondrial biogenesis and bioenergetics. In this study, the interventions stimulated mitochondrial biogenesis and bioenergetics by regulation of the electron transfer chain (ETC).

The first intervention of interest is an application of a low power of light. Transcranial photobiomodulation (PBM) with low-levels of red to near-infrared (NIR) light (wavelengths 600–1100 nm) has been proposed as a non-invasive method of stimulation in brain disorders (Hamblin, 2016; Salehpour and Rasta, 2017). The procognitive benefits of this light-based intervention have been observed in animal models of Alzheimer’s disease (De Taboada et al., 2011) and aging (Salehpour et al., 2017), and in patients with acute stroke (Hamblin, 2017), traumatic brain injury (TBI) (Naeser et al., 2014; Morries et al., 2015), and dementia (Berman et al., 2017; Saltmarche et al., 2017). Absorption of light by mitochondrial complex IV, the cytochrome c oxidase (CCO) and subsequent increase in ATP production have been proposed as a main mechanism of action of PBM therapy (de Freitas and Hamblin, 2016), thought to act by augmentation of cerebral bioenergetics (Dong et al., 2015), inhibition of neuroinflammation (Lee et al., 2016; Salehpour et al., 2018b), and increase of antioxidant activity (Lu et al., 2017).

The second potential intervention of interest is administration of Coenzyme Q_10_ (CoQ_10_), also known as ubiquinone, that transfers electrons from complexes I and II to complex III (Dallner and Sindelar, 2000). CoQ_10_ supplementation is held to improve mitochondrial function by promotion of ATP production (Hargreaves, 2014) and mitochondrial biogenesis (Noh et al., 2013; Khorrami et al., 2014). In neurons, the action of CoQ_10_ has been shown to protect mitochondria and lipid membranes (Somayajulu et al., 2005), as has the beneficial effect of CoQ_10_ supplementation on the inflammatory response of endothelial cells (Gao et al., 2012), and the neuroprotective properties of CoQ_10_ against learning impairment in aged animals (Mcdonald et al., 2005; Shetty et al., 2013).

Here, we test the hypothesis that the added effects of senescence modeled by D-galactose in mice followed by BCCAO can be reversed by the beneficial effects on mitochondria of NIR light PBM and CoQ_10_ supplementation (alone or in combination), as evidenced by the potential procognitive and neuroprotective actions of the interventions.

## Materials and Methods

### Animals and Experimental Groups

According to the research design, 90 adult male BALB/c mice, 8–10-weeks-old and weighing 20–25 g, were purchased from Laboratory Animal Center of Tabriz University of Medical Sciences (TUOMS) Tabriz, Iran. Mice were socially housed in standard cages (five/cage), kept under controlled conditions at the temperature of 24 ± 2°C on a 12/12 h light and dark cycle, and fed standard pellet food with tap water *ad libitum*. The experiments were performed under an approved protocol of TUOMS Ethics Committee (No: IR.TBZMED.REC.1396.576) and were in accordance with NIH guidelines. After a week of adaptation, mice were randomly divided into two groups: control (*n* = 15) and the artificially aged (AA) (*n* = 75). The control group received normal saline (NS) (0.9% NaCl, 0.2 ml/mice) and the AA group received D-galactose (500 mg/kg/daily) (Sigma, St. Louis, MO, United States) by subcutaneous (s.c.) injection once per day for 6 weeks (Salehpour et al., 2017). At the end of the sixth week, mice in the AA group were divided into two groups: AA either separately (*n* = 15) or followed by transient global brain ischemia (GBI) (*n* = 60). The AA mice received sham surgery. Ischemic AA mice were then subdivided into four groups that received (I) sham PBM + vehicle gavage (AA+GBI), (II) sham PBM + CoQ_10_ gavage (CoQ_10_), (III) real PBM + vehicle gavage (PBM), and (IV) real PBM + CoQ_10_ gavage (PBM + CoQ_10_) (*n* = 15 per group).

We compared a vehicle-treated group of AA mice to a vehicle-treated control group (AA vs. control), and we compared a vehicle and sham-laser treated group of AA mice that we further stressed with transient GBI to the vehicle-treated group of AA mice (AA+GBI vs. AA). Three globally ischemic AA groups then received three different therapeutic interventions (CoQ_10_, PBM, or PBM+CoQ_10_) that we compared to a globally brain ischemic group of AA mice (AA+GBI) given only sham treatments (CoQ_10_, PBM, or PBM+CoQ_10_ vs. AA+GBI).

**FIGURE 1:**
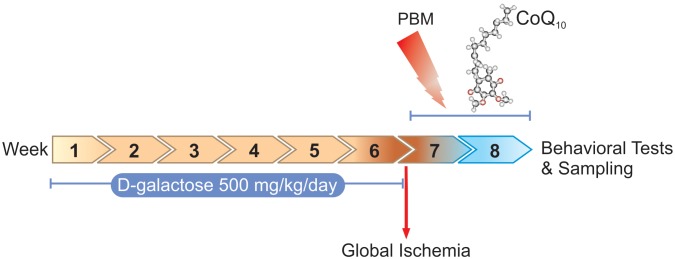
Timescale of D-galactose administration, global ischemia induction, PBM and/or CoQ_10_ treatments, behavioral tasks, and sampling.

Figure 1 shows the study design including procedures and time periods.

### Surgery

We induced transient global cerebral ischemia with the BCCAO method as previously described (Soares et al., 2016). Briefly, animals were anesthetized with inhalation of isoflurane (4%) and intraperitoneal injection of xylazine (4 mg/kg) and maintained with 1.5% isoflurane in 70% nitrous oxide and 30% oxygen through a plastic facemask. Rectal temperature was maintained at 37°C with a heating blanket and monitored during operation. A midline anterior incision was made in the neck to expose the both common carotid arteries. Each artery was encircled with 3-0 silk thread and the vagus nerve separated. Transient global cerebral ischemia was induced by BCCAO for 20 min using microaneurysm clips (Aesculap, Germany) and CBF reduction was confirmed by laser Doppler flowmeter (LDF) (LaserFlo, Vasamedics Inc., St Paul, MN, United States). Then, anesthesia was turned off, the skin incision was sutured, and the mice were placed in a recovery cage. For sham surgery, the AA group was exposed to the same anesthetic and surgical procedures without occlusion of the carotid arteries.

### Treatments

We performed the PBM therapy with a NIR laser (Thor Photomedicine, Chesham, United Kingdom) at 810 nm wavelength, 200 mW maximum output power, and 6.66 W/cm^2^ irradiance with a spot size of 0.03 cm^2^. The laser operated at 10-Hz PW frequency on at 88% duty cycle. For each irradiation session a total energy of 1 J (fluence of 33.3 J/cm^2^) was delivered to the head (Salehpour et al., 2017). For transcranial PBM therapy, each mouse was held firmly and the laser probe was positioned over the midline of the dorsal surface of the head in region between eyes and ears. The CoQ_10_ was purchased from Sigma-Aldrich (United States). The CoQ_10_ group received the treatment (500 mg/kg/day) via gavage (Schilling et al., 2001). CoQ10 was dissolved in soybean oil as a vehicle. The combination group received the same daily dose of CoQ_10_ and treated with PBM 2 hr before the gavage.

The AA+GBI group received oral vehicle and underwent identical PBM therapy procedures except that the laser device remained inactive. All oral administrations had a volume of 8 ml/kg body weight. All treatments were applied once a day for 14 consecutive days.

### Behavioral Assessments

#### Neurological Impairment

In order to evaluate neurological performance, mice were subjected to neurological severity score testing (NSS) on the day before starting the learning and memory tasks. The motor ability, balance, reflexes, and alertness of the animals were assessed as different aspects of neurological outcome (Beni-Adani et al., 2001). The scale ranges from 0 to 10. The intact control mice were given the score of zero (0), whereas a score of 10 reveals greatest neurological impairment. All behavioral testing was carried out by one person blinded to the experimental groups.

#### Barnes Maze Task

The spatial learning and memory abilities were evaluated using a Barnes task as described previously (Salehpour et al., 2017). The maze consisted of a black circular platform 100 cm in diameter with 20 evenly spaced holes located 3.0 cm from the perimeter. A movable rectangular escape box (20 × 15 × 5 cm) was located under the escape hole. The maze was placed in a quiet room where spatial cues (four basic shapes: triangle, square, circle, and horizontal rectangle) were located on the walls. Mice were encouraged to escape by a buzzer (80 dB white noise). The task procedure had three sessions including adaptation, training, and a probe trial, and took 5 days. On first day, each mouse was habituated to the task platform by gently guiding the mouse from the maze center to the target hole. The mouse was allowed to remain undisturbed in the escape box for 1 min after entering it. Each mouse then underwent 4 days of training with four 3-min trials per day, each separated by a 3-min interval. 24 h following the last training trial, a probe trial was performed, that is, the escape box was removed and the mouse was allowed to explore the maze freely for 3 min. Between trials, the maze was cleaned with 70% alcohol to remove odor cues. Trials were recorded using a digital camera mounted above the maze and the performance of mice was analyzed using a video tracking program Etho Vision^TM^ (Noldus, Netherlands). The performance during the training trials was evaluated by the latency time to find the target hole. Additionally, the time spent in the target quadrant, and correct/wrong relative time (time spent around the holes) during the probe session was calculated.

#### Lashley III Maze

Spatial reference memory and learning were evaluated using the Lashley III maze task, which is based on navigation with visual and food cues (Matzel et al., 2003). Briefly, the lateral walls and ceiling of the maze were constructed from black or transparent Plexiglas, respectively. The maze consisted of three separable parts including start box, maze arms, and goal box. The maze arms had four lanes that were evenly spaced along the maze. The small cup containing sugar reward was located in the goal box as motivation. Before the start of the test, mice were fasted for 12 h. Mice were moved to the testing room for 30 min prior to placement in the maze. At the beginning of testing, each mouse was placed in the start box for 15 s and then the Plexiglas sliding door was opened to allow the animal to explore the four alleys of the maze for 6 min. The trial is completed when the animal has entered the goal box at the end of the maze. Mice were trained once a day for 5 days and all experimental procedures were recorded using a digital camera and analyzed by a video tracking program Etho Vision^TM^ (Noldus, Netherlands). The learning index was measured as mean time in correct arms divided by total escape time during 5 days. The number of errors (i.e., a turn in an incorrect direction) and latency time to reach the goal box were also parameters of interest (Bressler et al., 2010).

#### What-Where-Which (WWWhich) Task

This task has been described in detail earlier (Salehpour et al., 2017) and is therefore described here briefly. The apparatus consisted of two different contexts, namely contexts 1 and 2, made of Plexiglas. All objects for testing were assembled from LEGO^®^ bricks. At the beginning of the task, each mouse was habituated to each context for 5 min for 1 day. After 24 h, as an exposure session, two different objects (A and B) were first located in the context 1 and the mouse was given 3 min to explore it. Then, the mouse was returned to the cage for 30 s and the locations of the objects were switched (B and A) in the context 2. Next, the mouse was placed in the context 2 and was allowed to explore it for 3 min. The test session was carried out after a 5 min interval, when the mouse encountered two copies of one of the objects (A or B) located in one of the two contexts (1 or 2) for 3 min. Exposure and test sessions were performed in 2 trials for 2 days with new objects for each day. The total observation time (sum of time spent exploring both novel and familiar objects) and locomotor activity were videotaped and data extracted using a video tracking program Etho Vision^TM^ (Noldus, Netherlands). The episodic-like memory was defined by the discrimination index (DI) as follows: DI = (N-F)/(N+F), where N and F represent the exploration time on the novel and familiar objects, respectively.

### Biochemical Assessments

#### Sampling and Mitochondrial Isolation

Mice were sacrificed with an overdose of ketamine (100 mg/kg) and xylazine (10 mg/kg), at 24 h after the last behavioral test. The whole brain (excluding cerebellum) was quickly taken out from the skull and placed in ice-cold isolation buffer. Then, brain tissues were cut into small pieces and homogenized in ice-cold extraction buffer. The samples were centrifuged at 600 ×*g* in 4°C for 5 min. The supernatant liquid was transferred into a new tube and centrifuged at 12,000 ×*g* in 4°C for 15 min. The pellet was resuspended in storage buffer. Protein levels were determined by Bradford method (Bradford, 1976).

#### Mitochondrial ROS Levels

For evaluation of the ROS level in brain mitochondria, the fluorescent dye, dichlorohydro-fluorescein diacetate (DCFDA), was used (Novalija et al., 2003). The mitochondria were incubated with 2-μM DCFDA at 37°C for 20 min. The fluorescence intensity was determined (λ_ex_ = 485 nm, λ_em_ = 530 nm) in a fluorescence microplate reader. The ROS levels were represented as fluorescence intensity and normalized to samples protein.

#### ATP Colorimetric Assay

All procedures were done according the colorimetric assay kit (MAK190, Sigma, United States). Briefly, the homogenized brain tissue was mixed with ATP assay buffer followed by development buffer and the absorbance was measured at 570 nm. A standard curve was used to obtain the ATP concentration and presented in units of μmol per g tissue.

#### Mitochondrial Activity

Mitochondrial labeling was performed using MitoTracker Probes (Cell signaling, United States). The MitoTracker Green (MTG) was directly added into mitochondrial suspension (100–400 nM) and incubated for 30 min. The fluorescence intensity was determined (λ_ex_ = 490 nm, λ_em_ = 516 nm) in a fluorescence microplate reader. The mitochondrial activity was represented as fluorescence intensity and normalized to sample protein content.

#### Western Blotting

Cytosolic and mitochondrial proteins of interest were analyzed by Western blot method as described previously (Sadigh-Eteghad et al., 2015). Briefly, proteins were separated using 12.5% polyacrylamide gel and transferred onto a poly-vinylidene-difluoride (PVDF) membrane (Roche, United Kingdom). Membranes were incubated with anti-SIRT1 (sc-15404), PGC1-α (sc-5815), NRF1 (sc-101102), TFAM (sc-166965), iNOS (sc-8310), TNF-α (ab6671), and IL-1β (sc-7884) primary antibodies in 1:500 concentration. Finally, membranes were placed in ECL prime Western blotting detection reagent (Amersham, United Kingdom). Signals were visualized by exposure to autoradiography film (Kodak, United States). Anti-GAPDH (sc-32233) and cytochrome c (sc-13156) antibodies were used for internal control of cytosolic and mitochondrial proteins, respectively. The signal intensity of each band was quantitated using ImageJ 1.62 software (National Institutes of Health, United States).

#### Statistics

Descriptive data were expressed as mean ± S.E.M. Comparison of different experimental groups was carried out by a one-way ANOVA followed by the *post hoc* Tukey test. All analyses were performed using Graph Pad Prism 6.01 (Graph Pad Software Inc., La Jolla, CA, United States). A *p*-value < 0.05 was considered statistically significant.

## Results

We designed the experiments to answer two main questions: Did the cerebral ischemia further exacerbate the effects of artificial aging, and did one or more of three hypothetically beneficial interventions reverse the effects of the aging exacerbated by ischemia? The design of the interventions yielded the assessments below, as applied to each group of mice.

### Behavioral Assessments

#### Neurological Severity

No mice in any experimental group exhibited any behavioral change, as determined by NSS test (data not shown).

#### Barnes Maze

##### Training session

**FIGURE 2:**
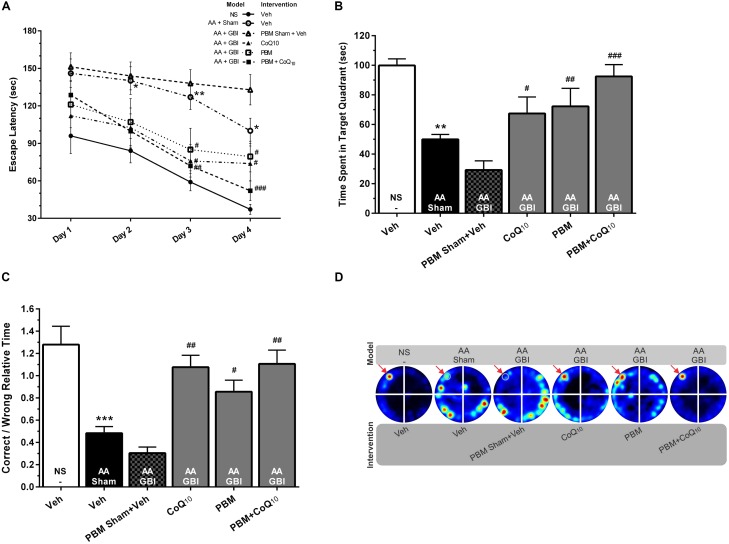
**(A)** Escape latencies in training sessions in different groups. Differences among groups were analyzed for each day. **(B)** Time spent in the target quadrant and **(C)** relative error time in probe sessions in different groups. **(D)** Corresponding heat maps display the combined traces of the mice from experimental groups during the probe session. Values represent the mean ± SEM, (*n* = 15). ^∗^*p* < 0.05, ^∗∗^*p* < 0.01, and ^∗∗∗^*p* < 0.001 compared with the NS (control). ^#^*p* < 0.05, ^##^*p* < 0.01, and ^###^*p* < 0.001 compared with the AA + GBI. AA, artificially aged; CoQ_10_, Coenzyme Q_10_; GBI, global brain ischemia; NS, normal saline; PBM, photobiomodulation; Veh, Vehicle.

The values of the animals’ velocity indices revealed no statistical differences among experimental groups for all four days of the training session. The one-way ANOVA followed by Tukey test showed that AA mice delayed the finding of the escape box on the 2nd (*p* < 0.05), 3rd (*p* < 0.01), and 4th (*p* < 0.05) day. There were no significant difference between the effects of artificial aging with or without subsequent ischemia in terms of latency (*p* > 0.05). The interventions, CoQ_10_ and PBM, both separately and together, significantly reduced the latency on the 3rd and 4th days of training (separately *p* < 0.05, together 3rd day *p* < 0.01, 4th day *p* < 0.001; Figure 2A).

##### Probe session

As shown in Figure 2B, the AA mice spent a significantly shorter time in the target quadrant (*p* < 0.01). No significant difference was observed between the aged mice with or without added ischemia in terms of time spent in the target quadrant (*p* > 0.05), but the subsequent treatment with CoQ_10_ and PBM separately or together caused treated mice to spend more time (*p* < 0.05, *p* < 0.01, and *p* < 0.001, respectively).

The correct-to-wrong relative times significant differed between the AA and control groups (*p* < 0.001). There were no significant differences between the AA and AA+GBI groups (*p* > 0.05), but the CoQ_10_ (*p* < 0.01), PBM (*p* < 0.05), and combination (*p* < 0.01) treatments significantly increased the correct-to-wrong relative times in the probe session (Figure 2C,D), although the effects of the three treatments did not differ.

#### Lashley III Maze

**FIGURE 3:**
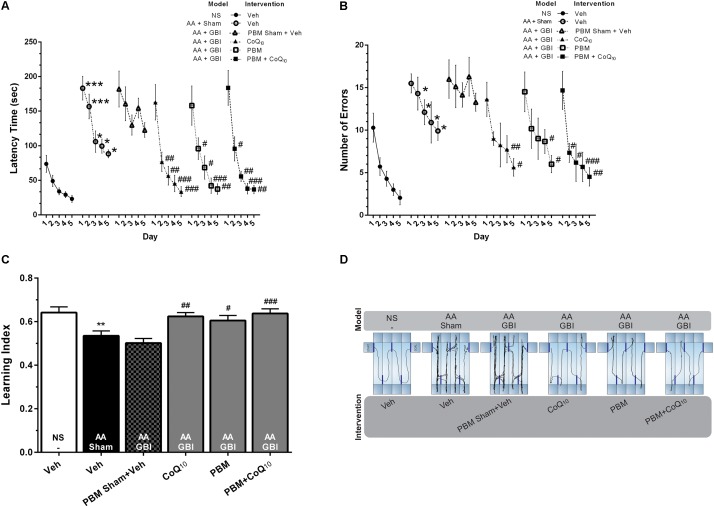
**(A)** Latency times and **(B)** number of errors in different groups. Differences among groups were analyzed for each day. **(C)** Learning index in different groups. **(D)** Corresponding sample tracks display the exploratory patterns during the last day of test. Values represent the mean ± SEM, (*n* = 15). ^∗^*p* < 0.05, ^∗∗^*p* < 0.01, and ^∗∗∗^*p* < 0.001 compared with the NS (control). ^#^*p* < 0.05, ^##^*p* < 0.01, and ^###^*p* < 0.001 compared with the AA + GBI. AA, artificially aged; CoQ_10_, Coenzyme Q_10_; GBI, global brain ischemia; NS, normal saline; PBM, photobiomodulation; Veh, Vehicle.

As shown in Figure 3A,D, the mean latency to reach the goal box was significantly higher in the AA group compared to the control group on the 1st and 2nd (for both, *p* < 0.001), 3rd, 4th, and 5th (for all, *p* < 0.05) days. There were no significant differences between the AA and AA+GBI groups in terms of mean latency (*p* > 0.05). The latency time on the 2nd, 3rd, 4th, and 5th days of training were significantly decreased after CoQ_10_ (*p* < 0.01, *p* < 0.01, *p* < 0.001, and *p* < 0.001) and PBM treatments (*p* < 0.05, *p* < 0.05, *p* < 0.001, and *p* < 0.01), respectively. In addition, the combination group showed a significant decrease in the latency on the 2nd (*p* < 0.05), 3rd (*p* < 0.01), 4th (*p* < 0.001), and 5th (*p* < 0.01) days.

The mean value of the number of errors was significantly higher in the AA group on days 2–5 days (for all, *p* < 0.05). There were no significant differences between the AA and AA+GBI groups in terms of the number of errors (*p* > 0.05). The number of errors on the 4th and 5th days of training, respectively, were decreased in CoQ_10_ (*p* < 0.01, and *p* < 0.05) and PBM (for both, *p* < 0.05) groups. In addition, the combination group showed a significant decrease in the number of errors on the 2nd and 3rd (for both, *p* < 0.05), 4th (*p* < 0.001), and 5th (*p* < 0.01) days (Figure 3B).

As shown in Figure 3C, the AA mice exhibited a lower learning index compared to control mice (*p* < 0.01). No significant difference was observed between the AA and AA+GBI groups in terms of learning index (*p* > 0.05). On the other hand, CoQ_10_ (*p* < 0.01), PBM (*p* < 0.05), and combination (*p* < 0.001) treatments significantly increased mean values of the learning index during 5-day trials.

#### What-Where-Which (WWWhich) Task

**FIGURE 4:**
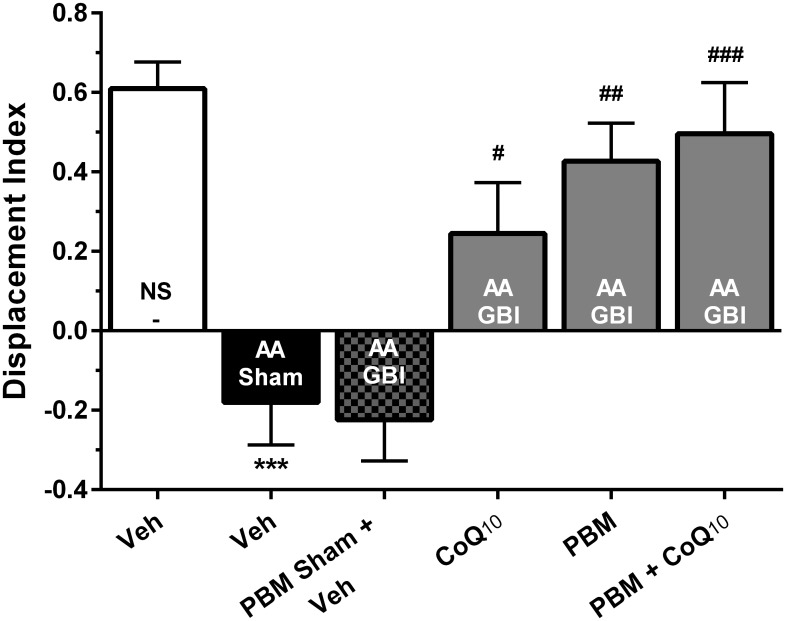
Displacement index (DI) in different groups. Each bar represents the mean ± SEM, (*n* = 15). ^∗∗∗^*p* < 0.001 compared with the NS (control). ^#^*p* < 0.05, ^##^*p* < 0.01, and ^###^*p* < 0.001 compared with the AA + GBI. AA, artificially aged; CoQ_10_, Coenzyme Q_10_; GBI, global brain ischemia; NS, normal saline; PBM, photobiomodulation; Veh, Vehicle.

There were no significant differences in locomotor activity and total observation time among groups (for both, *p* > 0.05) (data not shown). Artificial aging significantly impaired the DI in comparison to the control group (*p* < 0.001), while no significant difference was observed between the AA and AA+GBI groups (*p* > 0.05). The CoQ_10_, PBM, and combination groups notably increased the DI value as a recognition performance (*p* < 0.05, *p* < 0.01, and *p* < 0.001, respectively) (Figure 4).

### Biochemical Assessments

#### Mitochondrial ROS

**FIGURE 5:**
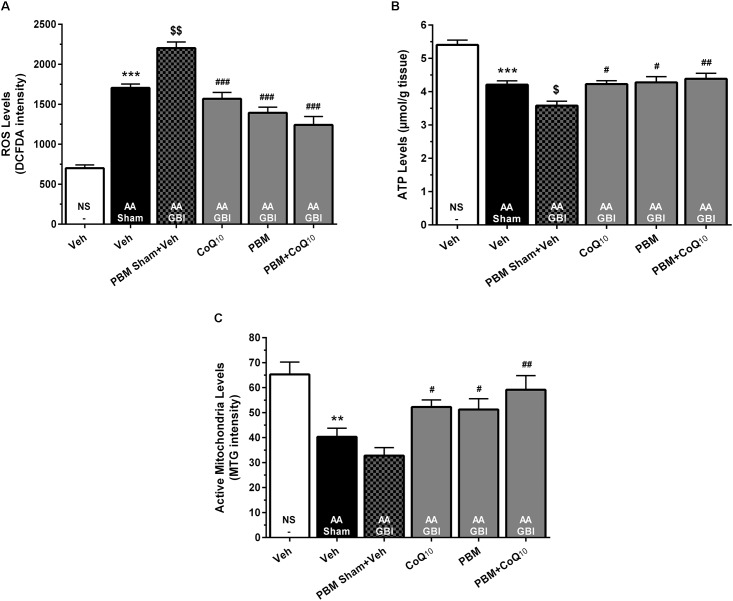
**(A)** DCFDA intensity as mitochondrial ROS levels, **(B)** ATP content, and **(C)** MitoTracker Green (MTG) intensity as mitochondrial activity in brain tissue of different groups. Each bar represents the mean ± SEM, (*n* = 8). ^∗∗^*p* < 0.01 and ^∗∗∗^*p* < 0.001 compared with the NS (control). ^$^*p* < 0.05 and ^$$^*p* < 0.01 compared with the AA.^#^*p* < 0.05, ^##^*p* < 0.01, and ^###^*p* < 0.001 compared with PBM- AA + GBI. AA, artificially aged; CoQ_10_, Coenzyme Q_10_; GBI, global brain ischemia; NS, normal saline; PBM, photobiomodulation; Veh, Vehicle.

The cerebral mitochondrial ROS levels rose markedly in the AA group (*p* < 0.001), and rose even further in the AA+GBI group (*p* < 0.01). Both PBM and CoQ_10_ treatments, alone and in combination, significantly returned the ROS values to the AA group level (for all, *p* < 0.001), as shown in Figure 5A.

#### ATP

The AA group mice had significantly reduced ATP levels (*p* < 0.001), with further significant reduction in the AA+GBI group (*p* < 0.05), and the CoQ_10_ (*p* < 0.05), PBM (*p* < 0.05), and combination (*p* < 0.01), treatments significantly returned the ATP levels to the AA group level (Figure 5B).

#### Active Mitochondria Levels

There was a lower active mitochondria level in the AA group (*p* < 0.01), with no further significant decline in the AA+GBI group (*p* > 0.05). The PBM, CoQ_10_ (for both, *p* < 0.05), and in combination (*p* < 0.01) separately, markedly and equally raised the active mitochondria level (Figure 5C).

#### Western Blotting

##### SIRT1

**FIGURE 6:**
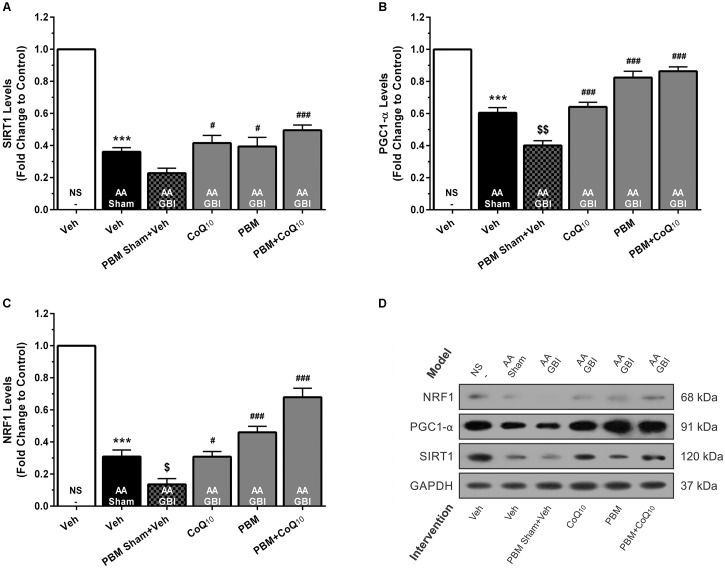
**(A)** SIRT1, **(B)** PGC1-α, and **(C)** NRF1 levels in brain tissue of different groups. Each bar represents the mean ± SEM, (*n* = 6). ^∗∗∗^*p* < 0.001 compared with the NS (control). ^$^*p* < 0.05 and ^$$^*p* < 0.01 compared with the AA. ^#^*p* < 0.05 and ^###^*p* < 0.001 compared with AA + GBI. **(D)** Representative images of SIRT1, PGC1-α and NRF1 protein levels detected by western blot. The GAPDH was used as an internal control. AA, artificially aged; CoQ_10_, Coenzyme Q_10_; GBI, global brain ischemia; NRF1, nuclear respiratory factor 1; NS, normal saline; PBM, photobiomodulation; PGC1-α, peroxisome proliferator-activated receptor gamma coactivator-1 alpha; SIRT1, silent mating-type information regulation 2 homolog 1; Veh, Vehicle.

SIRT1 levels were decreased in the AA group (*p* < 0.001), whereas no significant difference was observed between the AA and AA+GBI groups (*p* > 0.05). The CoQ_10_ (*p* < 0.05), PBM (*p* < 0.05), and combination (*p* < 0.001) treatments partially restored the SIRT1 levels (Figure 6A,D).

##### PGC1-α

Artificial aging significantly reduced the PGC-1α levels (*p* < 0.001). Also, the AA+GBI group exhibited significantly lower PGC-1α expression compared to the AA group (*p* < 0.01). Both CoQ_10_ and PBM groups had significantly raised PGC1-α levels (*p* < 0.001). In addition, the levels of PGC1-α were markedly upregulated in the combination group (*p* < 0.001; Figure 6B,D).

##### NRF1

Artificial aging significantly lowered NRF1 expression (*p* < 0.001) that further declined in the AA+GBI group (*p* < 0.05). The reductions were reversed by the CoQ_10_, PBM, and combination treatments (*p* < 0.05, *p* < 0.001, and *p* < 0.001, respectively), as shown in Figure 6C,D.

##### Cytosolic TFAM

**FIGURE 7:**
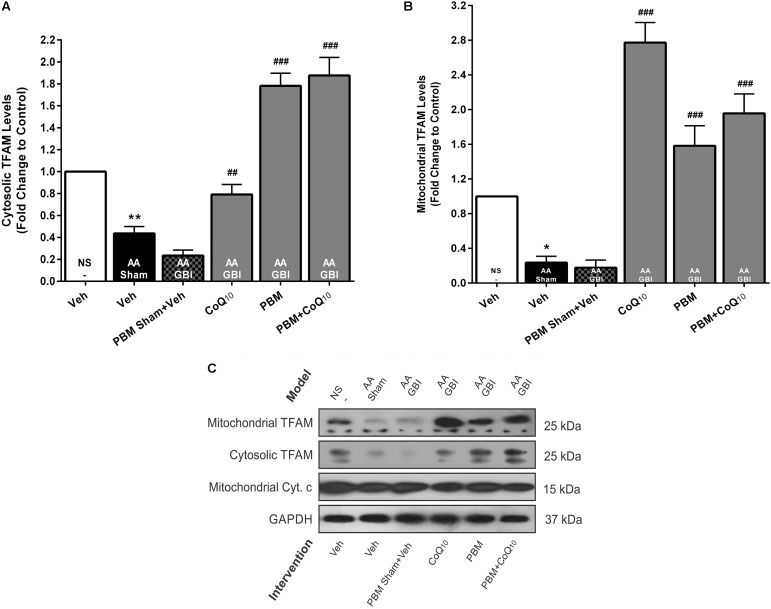
**(A)** Cytosolic and **(B)** mitochondrial TFAM levels in brain tissue of different groups. Each bar represents the mean ± SEM, (*n* = 6). ^∗^*p* < 0.05 and ^∗∗^*p* < 0.01 compared with the NS (control). ^##^*p* < 0.01 and ^###^*p* < 0.001 compared with AA + GBI. **(C)** Representative images of cytosolic and mitochondrial TFAM protein levels detected by western blot. The GAPDH and Cyt. c were used as cytosolic and mitochondrial internal controls, respectively. AA, artificially aged; CoQ_10_, Coenzyme Q_10_; Cyt. c, cytochrome c; GBI, global brain ischemia; NS, normal saline; PBM, photobiomodulation; TFAM, mitochondrial transcription factor; Veh, Vehicle.

Cytosolic TFAM was decreased in the AA group (*p* < 0.01), whereas no significant difference was observed between the AA and AA+GBI groups (*p* > 0.05). Although CoQ_10_ treatment partially increased cytosolic TFAM levels (*p* < 0.01), this increase was much more pronounced in both PBM and combination treatment groups (for both, *p* < 0.001) (Figure 7A,C).

##### Mitochondrial TFAM

As shown in Figure 7B,C, mitochondrial TFAM was decreased in the AA group (*p* < 0.05), whereas no significant difference was observed between the AA and AA+GBI groups (*p* > 0.05). However, both PBM and CoQ_10_ treatments, alone or in combination, remarkably increased mitochondrial TFAM levels (for all, *p* < 0.001).

##### iNOS

**FIGURE 8:**
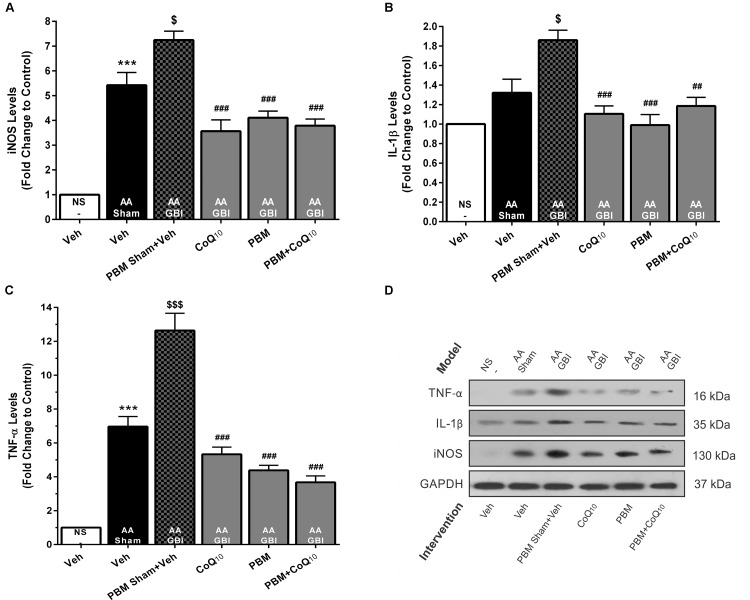
**(A)** The iNOS, **(B)** IL-1β, and **(C)** TNF-α levels in brain tissue of different groups. Each bar represents the mean ± SEM, (*n* = 6). ^∗∗∗^*p* < 0.001 compared with the NS (control). ^$^*p* < 0.05 and ^$$$^*p* < 0.001 compared with the AA.^##^*p* < 0.01 and ^###^*p* < 0.001 compared with AA + GBI. **(D)** Representative images of iNOS, IL-1β, and TNF-α protein levels detected by western blot. The GAPDH was used as an internal control. AA, artificially aged; CoQ_10_, Coenzyme Q_10_; GBI, global brain ischemia; IL-1β, interleukin-1β; iNOS, inducible NO synthase; NS, normal saline; PBM, photobiomodulation; TFAM, mitochondrial transcription factor; TNF-α, tumor necrosis factor-α; Veh, Vehicle.

The artificial aging caused a significant increase in iNOS levels (*p* < 0.001) that was further pronounced in the AA+GBI group compared to the AA group (*p* < 0.05). Both the PBM and CoQ_10_ treatments, alone and in combination, significantly reversed the effect on the iNOS levels (for all, *p* < 0.001) (Figure 8A,D).

##### IL-1β

As shown in Figure 8B,D, the AA group did not show a significant increase in IL-1β levels (*p* > 0.05). However, the AA+GBI group displayed significantly elevated IL-1β levels compared to the AA group (*p* < 0.05). On the other hand, the CoQ_10_ (*p* < 0.001), PBM (*p* < 0.001), and combination (*p* < 0.01) treatments showed significantly reduced IL-1β expression levels.

##### TNF-α

As shown in Figure 8C,D, a remarkable increase was observed in cerebral TNF-α levels in the AA group (*p* < 0.001). Also, a strong increase in TNF-α levels was observed in the AA+GBI group compared to the AA group (*p* < 0.05). However, both PBM and CoQ_10_ treatments, alone or in combination, significantly decreased TNF-α expression levels (for all, *p* < 0.001).

## Discussion

Here, we demonstrated significant and substantial improvements in neurobehavioral and molecular variables, including cerebral bioenergetics and mitochondrial biogenesis, as well as the neuroinflammatory response to artificial aging, in mice subjected to a model of transient global ischemia, by the PBM or CoQ_10_ treatments, or both.

Impaired mitochondrial function and inflammatory responses are considered crucial steps in the mechanisms involved in the model of brain aging induced by D-galactose (Shan et al., 2009; Kumar et al., 2010). In previous studies, aged animals were shown to be more sensitive to cerebral injury induced by an ischemic event (Canese et al., 1998; Xu et al., 2008), and transient BCCAO is known to induce an ischemia-reperfusion injury associated with oxidative damage to cortical neurons (Yabuki and Fukunaga, 2013).

Mitochondria are the principal organelles responsible for ATP generation and neuronal energy homeostasis (Onyango et al., 2010). Injured cerebrovascular endothelium in contrast is considered to be both source and target for ROS (Strasser et al., 1997). Age-related over-production of ROS results in endothelial apoptosis through oxidation of mitochondrial proteins and damage to and mutations in mitochondrial DNA (Dai and Rabinovitch, 2009). Likewise, a large body of evidence demonstrates that increased mitochondrial ROS generation follows chronic administration of D-galactose (Pratchayasakul et al., 2017), simulating the ROS generated in aging brain. Furthermore, transient reductions of CBF can deprive the brain of O_2_ and glucose, further exacerbating mitochondrial dysfunction (Sakoh et al., 2000).

Cerebral ischemia is prevalent in the aged population (Chen et al., 2010). In addition, susceptibility to oxidative stress caused by ischemic injury typically is higher in the aging brain compared to the young healthy brain (Lucas and Szweda, 1998). Therefore, oxidative damage induced by ischemic brain insults in the aged population plays a cardinal role in the mitochondrial dysfunction and subsequent cognitive decline in these subjects (Fukui et al., 2002).

Based on the preliminary study using 810 nm laser light in BALB/c mice (Salehpour et al., 2017), the fluence that reaches the cortical surface is only 8 J/cm^2^, when 33.3 J/cm^2^ of laser fluence is delivered to the scalp. In terms of light penetration through the brain tissues of BALB/c mice, it has been reported that approximately 3% of 810 nm laser photons reach a depth in the brain of 5 mm, the distance from skull surface to substantia nigra compacta (SNc) region (Reinhart et al., 2017). Therefore, it could be presumed that in this study 0.25 J/cm^2^ of NIR light reaches the brainstem, which is in the biostimulatory range for PBM therapy (Sharma et al., 2011; Moro et al., 2014).

A main mechanism of the beneficial effect of NIR PBM therapy relies on the improvement of mitochondrial function by direct stimulation of the ETC (Salehpour et al., 2018d). In this context, Lapchak and de Taboada (Lapchak and De Taboada, 2010) reported an increase in ATP content following 808 nm laser therapy and proposed this mechanism as a possible explanation for the reduction of ischemic tissue and the behavioral improvements following experimentally induced stroke. Previous experiments with specific cognitive impairment models have shown that NIR PBM therapy can improve mitochondrial functions by increasing ATP (Dong et al., 2015; Salehpour et al., 2017) and mitochondrial membrane potential (MMP) (Salehpour et al., 2017), and decreasing the over-production of ROS (Dong et al., 2015; Salehpour et al., 2017, 2018c).

In the current study, NIR PBM therapy significantly affected mitochondrial dysfunction induced by global ischemia and D-galactose administration, as shown by increased ATP synthesis and suppressed ROS production. To enhance the therapeutic efficiency of PBM, we combined PBM with CoQ_10_, a critical cofactor in the mitochondrial ETC known to improve mitochondrial function. Four weeks of CoQ_10_ supplementation exerted neuroprotection by decreasing the infarct volume of an ischemic hemisphere in aged mice (Li et al., 2007). Oral administration of CoQ_10_ for 30 days improved brain bioenergetics and reduced the effects of transient cerebral ischemia on mitochondrial function as indicated by increased O_2_ consumption and ATP production (Horecky et al., 2011). It is known that CoQ_10_ acts as a potent free radical scavenger against cerebral ischemia-induced oxidative damage (Ostrowski, 2000). Thus, our results, along with others, confirm that CoQ_10_ supplementation rescues mitochondrial function and prevents brain damage produced by a model of cerebral ischemia applied to a model of aging in mice.

Altogether, data from the CoQ_10_ and PBM combination regimen demonstrated slightly better results than those of each separate treatment in terms of ATP and ROS levels. In this regard, Dong et al. (Dong et al., 2015) have shown that NIR (810 nm) PBM therapy in combination with lactate or pyruvate synergistically increased hippocampal ATP levels in a mouse model of TBI. However, results from ROS levels did not show any significant differences among combination and separate treatment groups (Dong et al., 2015).

**FIGURE 9:**
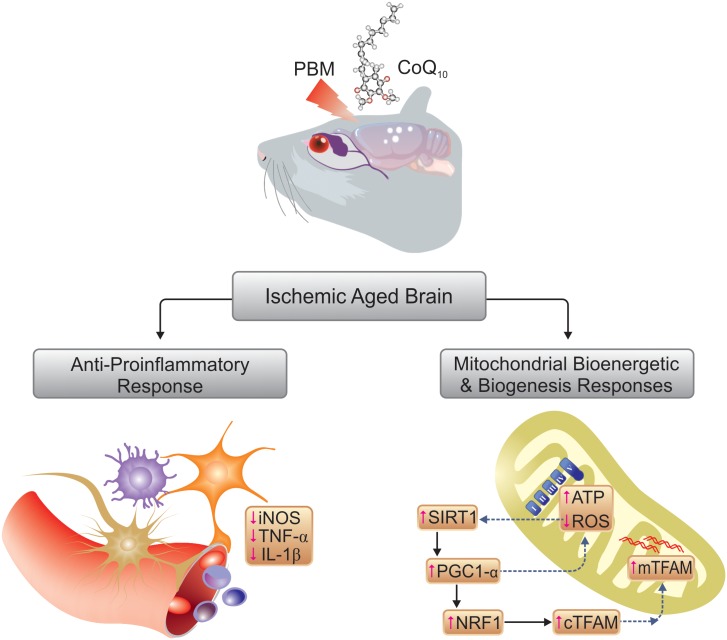
Schematic illustration of mechanisms involved in therapeutic effects of PBM and CoQ_10_.

Although an exact understanding of the cellular mechanisms underlying the suppression of cerebral overproduction of ROS after PBM therapy is still missing, the upregulation of total antioxidant capacity and some individual antioxidant enzymes [such as superoxide dismutase (SOD) and glutathione peroxidase (GPx)] may contribute to the beneficial effects (Lu et al., 2017; Salehpour et al., 2018c). Besides antioxidants, SIRT1 is linked to the oxidative stress response in cerebral ischemia (Raval et al., 2008). Cellular defenses attenuate mitochondrial oxidative damage, via activation of SIRT1 in cerebral ischemic injury (Yang et al., 2015). In addition, SIRT1 directly affects PGC1-α activity via phosphorylation and deacetylation (Cantó and Auwerx, 2009). PGC1-α is a transcriptional coactivator that upregulates the aforementioned antioxidant enzymes, SOD and GPx (St-Pierre et al., 2003). Studies have shown a key role of PGC1-α-mediated ROS metabolism in cerebral ischemia (Chen et al., 2011). In this regard, a rise in PGC1-α expression level has been proposed as a protective mechanism against ROS-mediated neuronal death (St-Pierre et al., 2006). The antioxidant effect of alpha-lipoic acid through the up-regulation of SIRT1-dependent PGC1-α expression has also been reported in a rat cerebral ischemia model (Fu et al., 2014). In our study, PBM and/or CoQ_10_ significantly increased SIRT1 and PGC1-α expression, and it seems that activation of SIRT1/PGC1-α pathway could be another possible explanation for observed reduction in ROS (Figure 9).

Total mitochondrial contents and the integrity of mitochondrial DNA both are adversely affected by cerebral ischemia because of the increase of oxidative stress (Chen et al., 2011). Mitochondrial biogenesis alters the size and number of individual mitochondrial units. Another essential function of PGC1-α, as a master activator of mitochondrial biogenesis, is regulation of mitochondrial content via transcription factors such as NRF1 and NRF2, which in turn upregulate mitochondrial gene expression, and TFAM can affect nuclear DNA (Hock and Kralli, 2009). For instance, down-regulation of PGC1-α in aluminum-treated rats along with a decrease of NRF1 and TFAM (which act downstream from PGC1-α) resulted in decrease of mitochondrial DNA copy number and mitochondrial abundance in the hippocampus and corpus striatum regions of the brain (Sharma et al., 2013). Moreover, SIRT1 could directly interact with PGC-1α and increase its activity and expression, leading to upregulation of mitochondrial gene expression (Nemoto et al., 2005). Therefore, activation of mitochondrial biogenesis, and thus the level of mitochondrial activity in neurons, has substantial therapeutic potential since it may improve cerebral bioenergetics in both ischemic brain insults and in general aging.

Although activation of mitochondrial biogenetic regulators after PBM therapy has been reported in muscle (Nguyen et al., 2014) and mesenchymal stem cells (Yin et al., 2017), there is no reported evaluation of stimulatory effects of NIR PBM therapy on neuronal mitochondrial biogenesis in the cerebral ischemic aging model. It has been demonstrated that 4 days of NIR laser irradiation (with dual wavelengths of 808+980 nm) can induce mitochondrial biogenesis by elevation of SIRT1 and PGC-1α in C_2_C_12_ muscle cells (Nguyen et al., 2014). Likewise, 660 nm light has been shown to increase the expression of mitochondrial biogenesis-associated genes including PGC-1α, NRF1, and TFAM in mesenchymal stem cells at 24 h post-irradiation (Yin et al., 2017). Increased transcription of NRF1 and TFAM through the upregulation of SIRT1 and PGC-1α expressions has also been proposed for aging rat skeletal muscle following 810 nm PBM therapy (Shaafi et al., 2014).

The present data showed that both PBM and/or CoQ_10_ could remarkably activate expression of genes, which are involved in mitochondrial biogenesis. Based on the findings from this preclinical research, we suggest that stimulation of the SIRT1/PGC1-α/NRF1/TFAM signaling pathway by NIR PBM and CoQ_10_ could regulate neuronal mitochondrial biogenesis, thereby providing neuroprotection against delayed cell death after cerebral ischemia, especially in aged brains (Figure 9). Taken together, mitochondrial function is linked to mitochondrial content that potentially can be improved by PBM therapy. Increased mitochondrial activity in the brain following 810 nm laser irradiation has been reported by this laboratory in animal models of cognitive impairment (Salehpour et al., 2017, 2018c), in line with the present results. Likewise, 2 weeks of CoQ_10_ treatment notably augmented the mitochondrial activity. Hence, the findings provide evidence that stimulation of neuronal mitochondrial biogenesis may be one of the key mechanisms for NIR PBM and CoQ_10_ to increase mitochondrial content and/or function, thus, attenuating cognitive impairment in a model of global cerebral ischemia superimposed on a model of aging.

It is believed that microglial cells activated in response to brain ischemic insults exacerbate cerebral injury by releasing pro-inflammatory TNF-α and IL-1β (Lakhan et al., 2009). The accelerated proliferation of glial and endothelial cells after an ischemic insult results in rapid infarct development in senescent animals that display age-dependent ischemic outcomes (Popa-Wagner et al., 2007). Decreased levels of TNF-α (De Taboada et al., 2011; Lu et al., 2017; Yang et al., 2018) and IL-1β (Moreira et al., 2009; De Taboada et al., 2011; Zhang et al., 2014; Lee et al., 2017; Lu et al., 2017) have been reported in specific animal brain injury models following transcranial PBM therapy. Pre-treatment with NIR PBM also resulted in significant reduction of cerebral TNF-α and IL-1β levels in ischemic mice (Lee et al., 2016). In accordance with the previous reports, our results showed increased inflammatory responses in a model of concurrent global ischemia superimposed on a model of aging in mice, while both PBM and/or CoQ_10_ partially, but significantly, attenuated the expression levels of TNF-α and IL-1β. Besides the proinflammatory cytokines, increased activity of iNOS in glia and infiltrating neutrophils leads to excessive amounts of free radicals following ischemic injury (Lakhan et al., 2009). The NO produced by iNOS within microglia and cerebrovascular endothelium is also involved in the deleterious inflammatory response occurring after ischemia (Zoppo et al., 2000). *In vitro* (Yang et al., 2010) and *in vivo* (Leung et al., 2002; Lee et al., 2016) studies support the idea that the anti-neuroinflammatory effects of transcranial PBM therapy may at least partly be due to its ability to modulate iNOS activity. In addition, CoQ_10_ treatment has been reported to attenuate hippocampal TNF-α levels in a pentylenetetrazole-induced cognitive dysfunction model (Bhardwaj and Kumar, 2016), and this finding is in good agreement with our data (Figure 9). Moreover, a study by Tsai et al. (Tsai et al., 2012) showed that CoQ_10_ could protect human endothelial cells from oxidative stress via decreasing iNOS expression.

To test whether PBM and/or CoQ_10_ protected against global ischemia-induced cognitive impairment performance in our aged-mouse model, we evaluated the animals in a test of spatial and episodic-like memories. Since the hippocampus plays a key role in the consolidation of memories related to spatial navigation, we subjected mice to the Barnes and Lashley III mazes as hippocampus-dependent spatial learning and memory tasks. The data showed that administration of D-galactose and global ischemia induction together significantly impaired spatial learning and memory, which is in line with previous reports (Xu et al., 2000; Sadigh-Eteghad et al., 2017; Salehpour et al., 2017). On the other hand, 2 weeks of PBM and/or CoQ_10_ treatments significantly reversed this deficiency. The procognitive benefits of NIR PBM therapy to spatial memory have been shown with the Morris water maze (De Taboada et al., 2011; Dong et al., 2015) and Barnes maze (Lu et al., 2017; Salehpour et al., 2017, 2018c) in different animal models. It is well established that the hippocampal bioenergetic capacity and neuronal survival are linked to spatial memory and learning (Sadowski et al., 2004).

In this study, we tentatively conclude that augmentation of brain bioenergetic capacity and the accompanying neuroprotective effects were reflected in improved learning and memory performances in both NIR PBM and CoQ10 treated mice. We observed that NIR PBM therapy notably improved the episodic memory of the D-galactose-treated mice in the BCCAO model of cerebral ischemia. This result is in line with previous reports of Lu et al. (Lu et al., 2017) and Salehpour et al. (Salehpour et al., 2017, 2018c) who demonstrated enhanced recognition memory after transcranial 810-nm laser therapy. Moreover, CoQ_10_ supplementation significantly rescued recognition memory as indicated by higher DI which is in line with other work (Llewellyn et al., 2015).

Since both chronic injection of D-galactose and subjection of animals to global cerebral ischemia result in systemic damage to the brain and nervous system, we examined the whole brain for possible neurochemical alterations. Furthermore, to test whether treatments protected against global ischemia-induced cognitive decline in the aged animal model, we subjected mice to the Barnes maze and Lashley III maze (as hippocampus-dependent spatial learning and memory tasks) and the WWWhich task (as a prefrontal cortex-dependent episodic memory task).

We note that the Red/NIR light propagation in the brain raises an important issue for purposes of treatment. Lambert-Beer’s law explains attenuation of photon propagation in biological media. Most recently, we showed that approximately 1% of the light incident onto the scalp surface reaches a 1-mm depth from the cortical surface, penetrating the dorsal hippocampus of the mouse, when a laser beam is focused on the bregma, (Salehpour et al., 2018a). Therefore, in the current study, we assumed that the cortical neurons and upper layers of the hippocampus received more photons than deeper structures (i.e., thalamus and hypothalamus). Given this, we also assumed that the neurochemical effects would be stronger if only the hippocampus had been sampled. However, further studies are needed to gain more insight into specific brain regions (i.e., hippocampus, prefrontal cortex, and amygdala) that are affected by PBM therapy of cognitive impairment.

Although the administration of CoQ_10_ affects brain function systemically and globally, the upper cortical layers are mainly affected by transcranial PBM therapy due to the aforementioned biophysical properties. Hence, the restricted light penetration is a potential interpretative limitation of the studies (brain and non-brain tissues) of possible synergistic effects of the combination of PBM therapy and pharmacotherapy strategies.

## Conclusion

We conclude that NIR PBM combined with CoQ_10_ had multiple mechanisms of action, targeting mitochondrial bioenergetics and biogenesis as well as anti-neuroinflammation in this model of ischemic brain damage in AA animals. The present study suggests that the PBM and CoQ_10_ treatments, alone or in combination, have procognitive effect in ischemic damage to aged brains of mice. It is noteworthy that the translation of the concepts of the current study in terms of models and conditions into humans is challenging. However, considering the number of elderly patients that sustain significant brain damage following a heart attack or ischemic stroke each year, it makes sense to test this relatively inexpensive and completely non-hazardous intervention (or combination of interventions) in humans. Further complementary preclinical and clinical studies are warranted for clarification of other potential applications in this area.

## Author Contributions

FS, FF, ME, JM, PK, and SR performed the experiments, interpreted the results, and wrote the first draft manuscript. SS-E and MF designed the experiments. SS-E, MH and AG critically interpreted data and critically revised and approved the manuscript.

## Conflict of Interest Statement

FS is a consultant for ProNeuroLIGHT LLC, Phoenix, AZ, United States. MH is on the following Scientific Advisory Boards: Transdermal Cap Inc., Cleveland, OH, United States; Photothera Inc., Carlsbad, CA, United States; BeWell Global Inc., Wan Chai, Hong Kong; Hologenix Inc. Santa Monica, CA, United States; LumiThera, Inc., Poulsbo, WA, United States; Vielight, Toronto, ON, Canada; Bright Photomedicine, São Paulo, Brazil; Quantum Dynamics LLC, Cambridge, MA, United States; Global Photon Inc, Bee Cave, TX, United States; Medical Coherence, Boston MA, United States; NeuroThera, Newark, DE, United States; JOOVV Inc., Minneapolis–Saint Paul, MN, United States; AIRx Medical, Inc., Pleasanton, CA, United States; FIR Industries, Inc., Ramsey, NJ, United States; UVLRx Therapeutics, Oldsmar, FL, United States; Ultralux UV Inc, Lansing, MI, United States; Illumiheal & Petthera, Shoreline, WA, United States; MB Laser Therapy, Houston, TX, United States. He has been a Consultant for: Lexington int., Boca Raton, FL, United States; USHIO Corp., Japan; Merck KGaA, Darmstadt, Germany; Philips Electronics Nederland B.V.; Johnson & Johnson Inc., Philadelphia, PA; Sanofi-Aventis Deutschland GmbH, Frankfurt, Germany. He is a Stockholder in Global Photon Inc., Bee Cave, TX, United States and Mitonix, Newark, DE, United States. AG serves on advisory boards of The Dana Foundation, United States, and the Velux Stiftung and Daylight Academy, Switzerland. As the owner of MINDexult CVR, Denmark, he gives regular lectures and teaching sessions to pharmaceutical companies. He is the President of Landsforeningen til Bekæmpelse af Hjernesygdomme, Denmark. The remaining authors declare that the research was conducted in the absence of any commercial or financial relationships that could be construed as a potential conflict of interest.
